# The efficacy of 2780 nm Er,Cr;YSGG and 940 nm Diode Laser in root canal disinfection: A randomized clinical trial

**DOI:** 10.1007/s00784-024-05563-z

**Published:** 2024-02-26

**Authors:** Sara Zakaria Fahim, Rami Maher Ghali, Ahmed A. Hashem, Mary Medhat Farid

**Affiliations:** 1 Faculty of Dentistry, Department of Oral Medicine, Periodontology, Diagnosis and Radiology, Future University, Cairo, Egypt; 2 Faculty of Dentistry, Department of Prosthodontics, Ain Shams University, Cairo, Egypt; 3 Ain Shams University, Faculty of Dentistry, Endodontic Department, Cairo, Egypt; 4 Cleveland Dental Institute, Cleveland, OH USA; 5 Faculty of Dentistry, Department of Oral and Maxillofacial, Radiology, Ain Shams University, Cairo, Egypt

**Keywords:** Lasers, Bacterial Load, Dental Pulp Diseases, Periapical Periodontitis

## Abstract

**Objectives:**

Effective disinfection of the root canals is the cornerstone of successful endodontic treatment. Diminishing the microbial load within the root canal system is crucial for healing in endodontically treated teeth. The aim of this study was to evaluate the effect of 2780 nm Er,Cr:YSGG and 940 nm diode lasers on the eradication of microorganisms from single-rooted teeth with asymptomatic apical periodontitis.

**Materials and Methods:**

Thirty participants conforming to the inclusion criteria were randomly divided into 3 groups according to the disinfection protocol used; Conventional group: 2.5% Sodium Hypochlorite (NaOCl) and 17% EDTA solution NaOCl/EDTA, Dual laser group: 2780 nm Erbium, chromium: yttrium scandium-gallium-garnet (Er,Cr:YSGG) laser and 940 nm diode laser Er,CrYSGG/Diode, and Combined group: 17% EDTA and 940 nm diode laser EDTA/Diode. Bacterial samples were collected before and after intervention. The collected data were statistically analyzed using Friedman’s test and Kruskal–Wallis test (*P* ≤ *0.05*).

**Results:**

The results of the study showed that both dual laser Er,CrYSGG/Diode and combined laser EDTA/Diode groups showed significantly less mean Log10 CFU/ml of aerobic and anaerobic bacterial counts than the conventional NaOCl/EDTA group.

**Conclusions:**

In this study we evaluated in vivo the bactericidal efficacy of three disinfection protocols for endodontic treatment of single-rooted teeth with apical periodontitis. The results indicated that both dual laser Er,CrYSGG/Diode and combined laser EDTA/Diode groups provide superior bactericidal effect compared to the conventional NaOCl/EDTA group.

**Clinical relevance:**

The integration of lasers into root canal disinfection protocols has demonstrated significant bacterial reduction which might promote healing and long-term success.

## Introduction

The ultimate goal of endodontic treatment is the creation of a sterile, bacteria-free environment inside the tooth as well as the periapical tissue and the surrounding bone to prevent or treat chronic periapical lesions [1]. Sodium hypochlorite (NaOCl) is the most commonly used irrigating solution in endodontic practice due to its ability to dissolve organic matter as well as its broad antimicrobial action [2]. Ethylenediaminetetraacetic acid (EDTA) is a chelating agent that is employed to remove calcium from dentine leaving a softened matrix. It also removes the smear layer associated with root canal instrumentation. and facilitates the mechanical preparation of the root canal [3]. Combining NaOCl and EDTA can be considered the standard method of endodontic cleaning.

However, conventional endodontic treatment is confronted with many challenges. First, it is limited by the morphological complexity of the root canal system, including lateral canals and accessory foramina especially in the apical area [4]. Second, 5% NaOCl is unable to eliminate bacteria that have penetrated deeper dentin layers, which may lead to recurrent endodontic lesions [5]. Thirdly, NaOCl is irritant to periapical tissues; a concentration of NaOCl over 0.5% is cytotoxic [6, 7]. Hence, prompting the need for new approaches to enhance endodontic disinfection.

Lately, laser therapy for root canal disinfection has gained attention. The goals for a laser-assisted root canal treatment are optimum removal of smear layer, deep penetration of laser energy into dentin, and intricate root canal structures to destroy microorganisms that can reach > 1000 µm inside dentin, as well as effective healing of damaged tissues [8, 9].

Er,Cr:YSGG laser belongs to the infrared Erbium family. It interacts with aqueous solutions and at a power of 3.5 W or more ablates efficiently dental hard tissue. Er,Cr:YSGG is used in endodontic treatment to remove the smear layer from the root canal walls because it exerts a limited penetration depth of 17 µm into the dentin [10]. Its bactericidal property depends on either instant evaporation of intracellular water or bacterial dehydration [11, 12]. In comparison with irrigation with EDTA, Er,Cr:YSGG laser has been consistently providing superior smear layer removal effectiveness [13].

940-nm diode laser is one of the near-infrared lasers that are highly absorbed by melanin and hemoglobin, and little absorbed by hydroxyapatite crystals or water. This property allows diode lasers to penetrate deep into dentine and exhibit its anti-bacterial effect [14, 15]. 940 nm Diode laser is used in endodontic treatment for disinfection of root canals. Diode laser has an intense antibacterial effect by causing changes in the bacterial cell wall and destroying the cell membrane^1^. Diode laser exerts a photo-thermal effect on the reachable bacteria. It also exerts a photo-disruptive effect on the unreachable bacteria; where immediate cell death might not occur; but rather sublethal damage occurs inhibiting the cell growth through the destruction of cell wall integrity and accumulation of denatured proteins causing the cessation of bacterial growth and consecutive cell lysis. This effect on bacteria occurs with very small doses of heat [16].

Diode laser uses a thin flexible 200 µm optical fiber to deliver its beam to the target area. The optical fiber can easily reach the apical third of the root canal, curved canals, and different anatomical areas that are difficult to access; eventually distributing the light consistently inside the root canal assuring a better photoreaction [1]. An additional advantage of diode laser is its extreme compactness, affordability, and ease of operation.

Well-reported in vitro studies have evaluated the bactericidal effect of Er,Cr:YSGG and diode laser [16–20]. One of these studies investigated the bactericidal potential of 2780-nm ER,CR:YSGG and 940-nm diode lasers. The findings revealed that the combined use of these lasers was more effective than either laser alone and is comparable to needle irrigation with sodium hypochlorite and EDTA [20].

An in vivo study assessed the results of laser-assisted endodontic treatment/ retreatment using 940-nm diode laser and EDTA in teeth with apical periodontitis. They concluded that 940-nm diode laser-assisted endodontic protocol is an effective replacement for conventional treatment that promotes faster healing of periapical lesions, requiring less chemical irrigation and systemic antibiotics [21].

further in vivo research used 2780 and 940 nm wavelengths for smear layer removal and achieved deep dentin disinfection respectively on two cases of relatively high endodontic complexity. Their 1-year follow-up supported the effectiveness of this treatment strategy [22].

Another study examined the safety of the combination of Er,Cr:YSGG and diode laser. They reported no unfavorable thermal variations on the external root surface. Thus, the aim of this study was to assess in vivo the impact of Er,Cr:YSGG/diode laser, and Diode/EDTA on the bacterial count in root canal treatment compared to conventional endodontic treatment in an evidence-based clinical trial. The null hypothesis being tested is that there will be no difference in total bacterial count reduction between the three groups [23].

## Methods

This study is a double-blinded, three-arm, randomized, clinical trial that was designed, reported, and written according to Preferred Reporting Items for Randomized Trials in Endodontics (PRIRATE) 2020 guidelines (Nagendrababu et al., 2020). The protocol was approved by the Faculty of Dentistry, Ain Shams University research ethics committee (approval number FDASU-REC ID041908). The trial design was registered at www.clinicaltrials.gov database with identifier number(NCT05964686).(28/07/2023).

https://clinicaltrials.gov/study/NCT05964686?term=NCT05964686&rank=1

## Sample size calculation

The sample size was calculated according to the results of the study by Wenzler et al.[24]. The standard deviation within groups was assumed to be 15%. The effect size (f) was 0.73 Using alpha (α) level of (5%) and Beta (β) level of (20%) i.e., power = 80%; the minimum estimated sample size was a total of 24 subjects (8 subjects per group). The sample size was increased to 10 subjects per group to compensate for a drop-out rate of 20%. Sample size calculation was performed using G*Power Version 3.1.9.2.

## Randomization

Thirty participants were randomly allocated using www.randomizer.org to 3 groups (n = 10). Randomization for this study was conducted on the Randomizer website on January 9th, 2022, at 4:00 PM. Patients referred to the outpatient endodontic clinic at the Faculty of Dentistry, Ain Shams University, between January and September 2022, were assessed for eligibility and subsequently assigned to groups in accordance with the pre-established randomization scheme.

## The inclusion criteria were as follows

Patients between 18 and 35 years old were included in this study if they had one single-rooted maxillary anterior tooth with necrotic pulp, closed apex, and asymptomatic apical periodontitis requiring root canal treatment. For inclusion in this study, the periapical lesion should have a periapical index score of 3 or 4 Ørstavik, et al.[25] The diagnosis of non-vital pulp was based on history-taking, clinical and radiographic examination.

The exclusion criteria were the presence of pain, swelling, a fistulous tract, or the presence of periodontal pockets more than 3 mm in the involved tooth. Patients who had received antibiotics during the last month, had previous root canal treatment in the related tooth, had any systematic disease, or had an allergy to NSAIDs were also excluded. The vulnerable groups, including pregnant females and mentally or physically disabled individuals, were not included. Patients who refused to participate in the study and those who were presented with technical difficulties during root canal treatment, including curved roots or complications during treatment such as broken files, were also excluded. The research goal and procedures were clearly explained to each patient before they signed the informed consent.

Patients were randomly distributed into 3 groups. Each participant was given a number from (1 to 30) using randomization software (www.randomizer.org). The root canal treatments were performed in a single visit by one investigator.

Antisepsis of the oral cavity was performed by rinsing for 1 min with 10 mL of 0.125% chlorhexidine gluconate mouthwash. Lidocaine topical anesthetic gel was used at the site of the injection. The tooth was anesthetized by buccal infiltration using (1:80,000 Arcaine, Aarge Pvt, India) local anesthetic solution using a side-loading cartridge aspirating syringe and a 30-gauge short needle.

Single-tooth isolation was performed using a suitable clamp and rubber dam. An antiseptic solution was used to disinfect the isolated tooth and surrounding rubber dam. All caries and/or coronal restorations were completely removed with sterile bur without exposure of the pulp chamber. The access cavity was prepared using sterile round carbide bur size #4. The patency of the canals was done using a stainless-steel hand K-file size 15. The root canal was irrigated with 1 ml of sterile saline solution. The root canals were scraped with H-file and irrigated with 1 ml of sterile saline solution. The first microbial samples (S1) were collected**,** the sample collection was adapted from the protocol previously described by Gomes et al. [26] to assess the initial colonizers of the root canals. Three sterile paper points were inserted into the root canal for 1 min each with pumping movements. Care was taken to avoid contact between the paper points and access cavity walls to prevent contamination. They were immediately inserted into sterile tubes containing a transport medium of thioglycolate and transferred to the microbiology lab.

Working length was measured using an apex locator (Root ZX mini, J. Morita, Japan) and then confirmed with an intraoral periapical radiograph to be 0.5–1 mm shorter than the radiographic apex. Root canals were mechanically prepared using ProTaper Next nickel-titanium rotary instruments (Dentsply Maillefer, Ballaigues, Switzerland) according to the manufacturer’s instructions until the X4 (40/06) master apical file. The patients were divided into 3 groups according to the disinfection protocol, as follows:

**Conventional group (NaOCl/EDTA):** 5 ml of 2.5% sodium hypochlorite for 1 min was used for irrigation between each file and the next using a 30-gauge side-vented needle reaching 2 mm short of the working length, then 5 ml of saline, followed by 1 ml of 5% sodium thiosulfate to neutralize the effect of NaOCl and finally 5 ml of 17% EDTA (Denteck, Zoetermeer, Netherlands) for 1 min was used at the end of the procedure to remove the smear layer. Thus, a total of 20 ml of NaOCl and a total of 5 ml of EDTA were used in the NaOCl-EDTA group.

**Dual laser group (Er,Cr:YSGG/Diode):** Er,Cr:YSGG intracanal laser irradiation was performed (ʎ = 2780 nm, 1.25 W source power, 20 Hz, 10% Air, 1% water) using a radial emitting tip RFT2 200 µm in diameter. 5 ml of 0.9% sterile saline was used for irrigation between laser applications. The irradiation protocol adopted for this study was 10 s irradiations followed by 10 s intervals, which constituted one lasing cycle. Each lasing cycle was performed 4 times for each root canal using the Er,Cr:YSGG laser to remove the smear layer.

That was followed by diode laser (Epic *X*™, BIOLASE Tech, Irvine, USA) for the activation process, diode laser (ʎ = 940 nm, 1W, source power) was utilized using the E2-14 tip, an endo 200 µm flexible laser tip with a 14 mm length, 1 mm short of the working length, in continuous mode, using the same irradiation protocol as previously mentioned [27].

**Combined group (EDTA/Diode):** 5 ml of 17% EDTA was used to remove the inorganic part of the smear layer. 5 ml of 0.9% sterile saline was used for irrigation. Disinfection of the root canal was done using the same diode laser machine and protocol.

Following the disinfection protocol, in all groups, sterile saline was placed in the canal, and then the canal was dried with 3 sterile ProTaper Next absorbent paper points corresponding to the same size as the master file. Each paper point was left for 1 min, and then the second microbial sample (S2) was collected to assess the colonization of bacteria and the status of the root canal just before obturation.

**Fig. 1:**
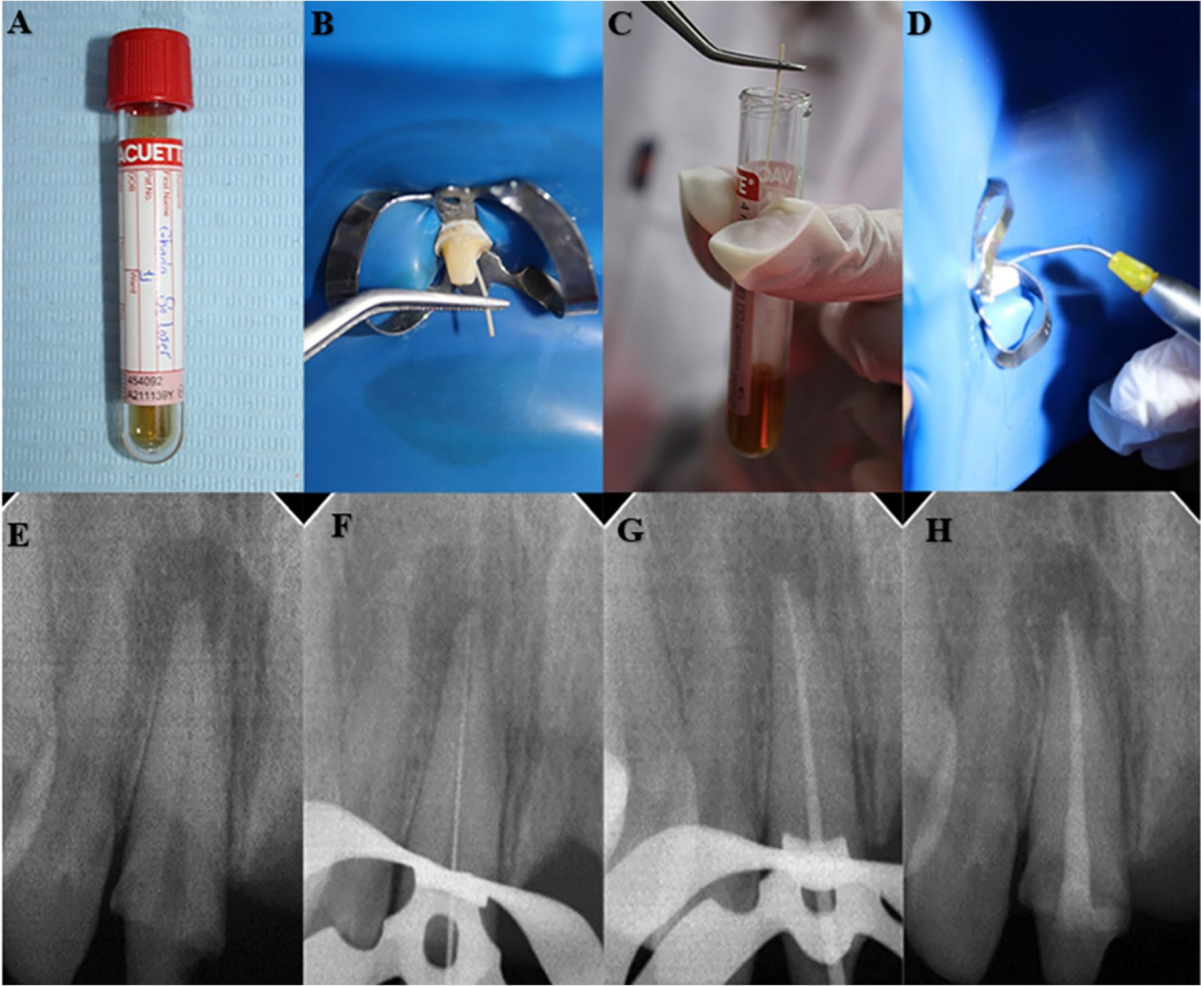
Sterile tube containing Thioglycolate, (B) Paper point is inserted inside the root canal, (C) The paper point is then inserted inside the sterile tube, (D) laser is applied inside root canal, (E) Preoperative periapical radiograph is taken, (F) Working length is confirmed with k-file #15, (G) Periapical radiograph is taken with the master apical cone in place, (H) Postoperative periapical radiograph is acquired

The root canals were obturated using the EQ-V system (Meta Biomed Korea) continuous wave condensation technique with proper selection of gutta-percha master cone corresponding to the same size as the master apical file and ADSEAL (Meta Biomed Korea) resin root canal sealer. Postoperative radiographs were taken to ensure proper obturation. The access cavity was sealed using a resin-modified glass ionomer. Some of the steps of the research are presented in Fig. 1.

## Microbiological analysis

### Bacterial count

Once the samples arrived at the microbiology lab, the tubes containing the thioglycolate (transport medium) (Thioglycollate broth U.S.P alternative, Oxoid microbiology product, England) with the paper points were placed in a microcentrifuge and vortexed for 30 s. One hundred µl aliquots of the vortexed samples were placed in a new sterile tube containing 1 ml of thioglycolate to obtain 1/10, 1/100, and 1/1000 concentrations to assess the microbial load of common aerobes and anaerobes found in each root canal.

### Aerobic bacterial culture

Fifty µl of these diluted samples were transferred to Brain Heart Infusion BHI (Oxoid microbiology product, England) agar plates and cultured under aseptic conditions, followed by incubation at 37 °C for 24 h for the aerobic bacteria. The number of bacterial colonies in each plate was counted and reported as colony-forming units per milliliter (CFU/ml).

### Anaerobic bacterial culture

The other 50 µl of these diluted samples were transferred to BHI agar plates under aseptic conditions. The agar plates were placed in an anaerobic sealed jar with Gas-Pak (Gas-Pak system) (Oxoid microbiology product, Basingstoke, Hants, England), and anaerobic indicator (Anaerobic indicator, BR0055B.Oxoid. Basingstoke, Hants, England) were incubated for 48 h at 37 °C. The number of bacterial colonies in each plate was eventually counted in each plate of the most diluted sample and reported as CFU/ml. Visible colonies were enumerated in each petri dish, and the number of colonies/plate was multiplied by the corresponding dilution factor and by 10 to determine the total CFUs/ mL of each specimen.

## Statistical Analysis

Numerical data were explored for normality by checking the distribution of data and using tests of normality (Kolmogorov–Smirnov and Shapiro–Wilk tests). Percentage reduction in bacterial counts data showed non-normal distribution. Data were presented as median, range, mean and standard deviation (SD) values. Friedman’s test was used to assess the changes in bacterial counts within each group after disinfection. The Kruskal–Wallis test was used to compare between the three groups. Dunn’s test was used for pair-wise comparisons when Kruskal–Wallis test or Friedman’s test is significant. The significance level was set at *P*  ≤*0.05*. Statistical analysis was performed with IBM SPSS Statistics for Windows, Version 23.0. Armonk, NY: IBM Corp.

## Results

**Fig. 2:**
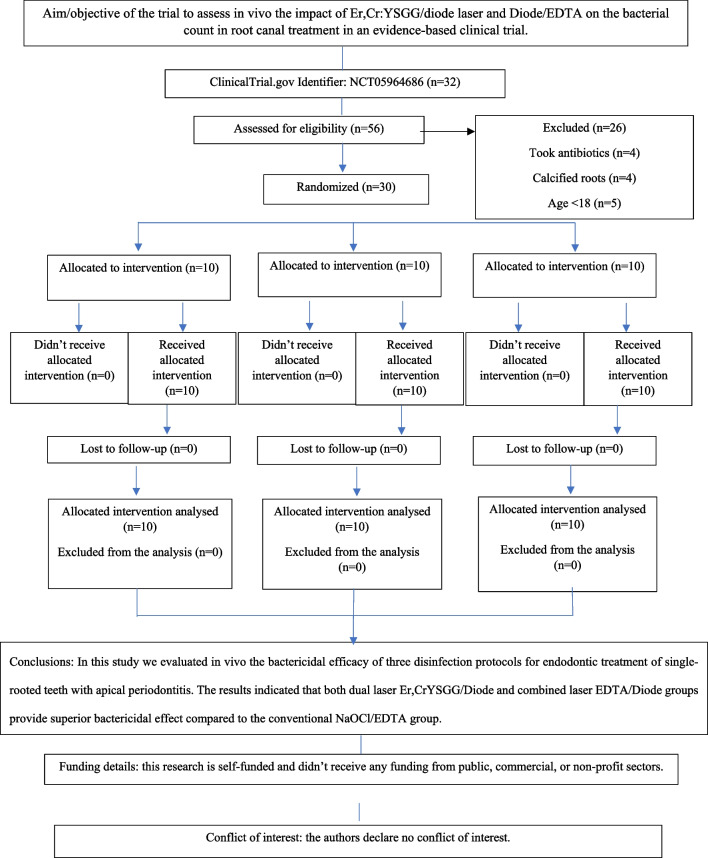
PRIRATE 2020 flowchart

A total of 56 patients were assessed for eligibility, 26 were excluded. The details are presented in a PRIRATE 2020 flow chart in Fig. 2. There were no dropouts in this study because the endodontic treatment was completed in one visit.

### Percentage reduction in bacterial counts

Percentage reduction was calculated as follows: [(Counts before application – After application) / Before application × 100]. There was a statistically significant difference between the three groups regarding the percentage reduction in aerobic and anaerobic bacterial counts. Pair-wise comparisons between the groups revealed no significant difference between Er,Cr;YSGG/Diode and EDTA/Diode groups; both showed significantly higher percentage reductions in aerobic and anaerobic bacterial counts than the NaOCl/EDTA group.

### Overall percentage reduction in bacterial counts

**Fig. 3:**
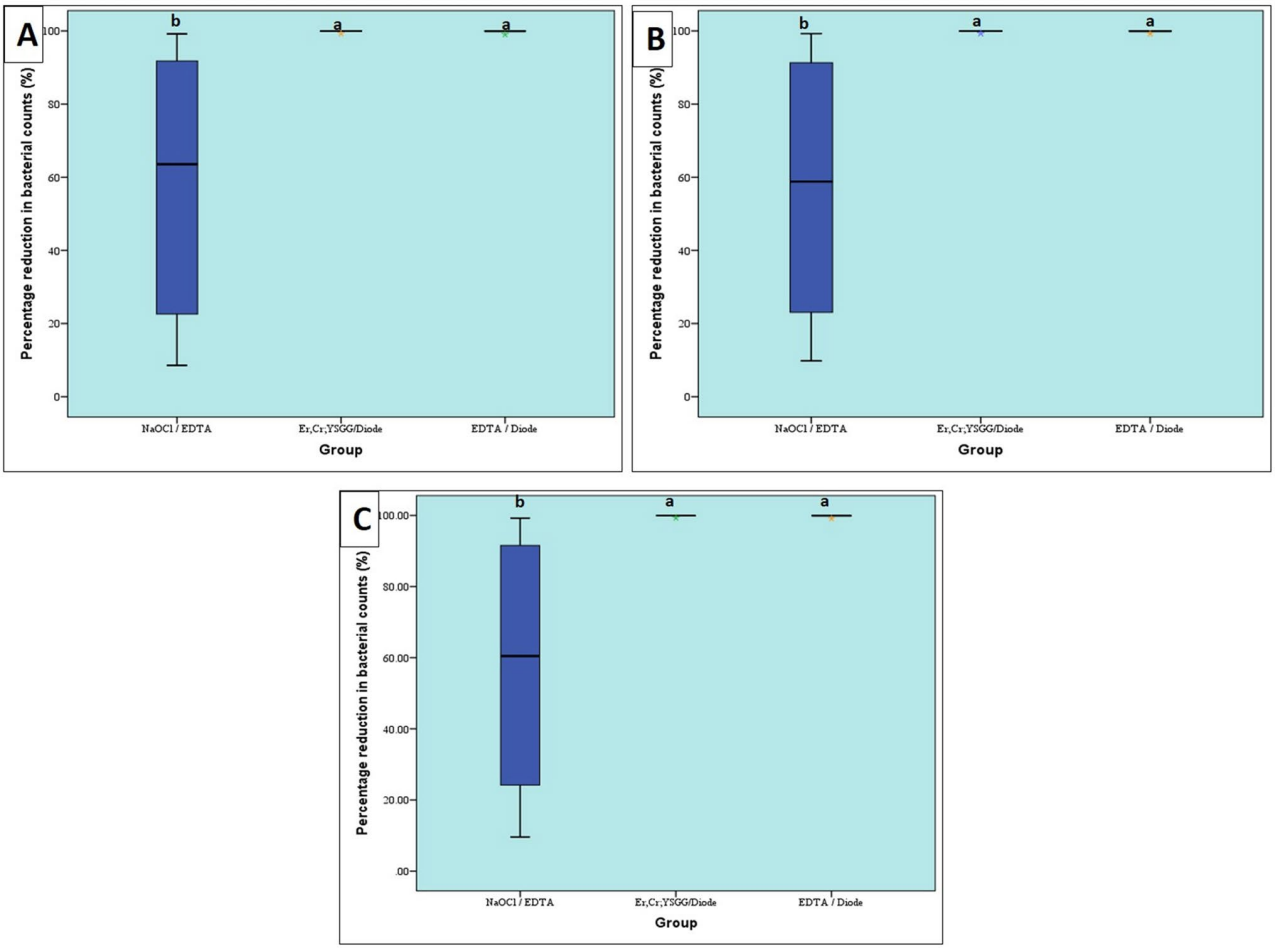
Box plot representing median and range values for percentage reduction in (A) aerobic, (B) anaerobic and (C) overall bacterial counts in the three groups. (Stars represent outliers)

A statistically significant difference was found between the three groups regarding the overall percentage reduction in bacterial counts. Pair-wise comparisons between the groups revealed that there was no significant difference between Er,Cr;YSGG/Diode, and EDTA/Diode groups; both showed higher percentage reduction in bacterial counts than NaOCl/EDTA group. The percentage reduction in aerobic, anaerobic bacterial counts and overall bacterial counts in the three groups are represented in Table 1, Fig. 3.

**Table 1:** Descriptive statistics and results of Kruskal–Wallis test for comparison between overall percentage reduction in bacterial counts (%) in the three groups

NaOCl / EDTA (n = 10)		Er,Cr;YSGG/Diode (n = 10)		EDTA / Diode (n = 10)		*P*-value	*Effect size (Eta squared)*
Median (Range)	Mean (SD)	Median (Range)	Mean (SD)	Median (Range)	Mean (SD)		
60.45 (9.62–99.25)^B^	57.6 (39.14)	99.97 (99.33–100)^A^	99.9 (0.2)	99.94 (99.16–99.99)^A^	99.88 (0.25)	< 0.001*	0.466

^*^: Significant at P ≤ *0.05, Different superscripts indicate statistically significant difference between groups*

## Discussion

The trial design of this prospective study was a double-blinded randomized clinical design since both the patients and the medical microbiologist who measured the bacterial count were blinded. The goal of randomization was to produce comparable groups in terms of general participants’ characteristics. Thus, eliminating any bias that may affect the relation between the interventions and the outcomes. The target population for this study included individuals between 18 and 35 years old. This age range was chosen due to the relative consistency of maxillary anterior teeth canal diameter and anatomy exhibited within this group. To further enhance sample homogeneity, patients with specific anatomical variations, such as open apices, were excluded from participation.

Patients under antibiotic therapy in the last month were excluded as this may reduce bacterial counts, inhibit and suppress bacterial growth which directly affect the colony count and impair the microbiological results, additionally antibiotic therapy cause disruption of the microbial flora acquiring a higher risk of developing postoperative infection and selecting for antibiotic resistant bacteria [28].

In the present study, dual laser group and combined laser groups resulted in less aerobic and anaerobic bacterial counts. Thus, the null hypothesis was rejected.

The success of endodontic treatment depends ultimately on the elimination of the microorganism, host response, and coronal seal of root canals [22]. Achieving long-term successful endodontic treatment without toxic chemical irrigants has always been a long-awaited goal of scientists. Hence the need for more sophisticated techniques to enhance disinfection like automated irrigation devices, ultrasonics [29], and lasers [30]. Combining NaOCl and EDTA represents the control group in this research because it is the standard method of endodontic disinfection. The Er,Cr;YSGG/Diode group was chosen to combine the intense antibacterial effect of diode laser together with the smear layer removal ability of Er,Cr:YSGG laser. The EDTA/Diode group represents a more economic merger, where the affordable diode laser with its antimicrobial effect is combined with 17% EDTA as a cost-effective chelating agent to remove the smear layer with minimal demineralizing effect [31] instead of the expensive Er,Cr:YSGG laser.

Root canal treatments for the three groups were completed in a single visit. According to the systematic review by Manfredi et al. [32] there is no evidence to suggest that single-visit or multiple-visit root canal treatment is better than the other. On the other hand, there is a tendency for clinicians to perform single-visit endodontic treatment recently owing to the benefits such as not requiring additional anesthetic injections, no need to replace the rubber dam or intracanal medication, absence of inter-visit leakage, loss of temporary seal, or any of the accidents that can and do occur between the visits [33]. Similarly, Özcan et al.[34] concluded that single-visit root canal treatment may be a strong alternative to multi-visit treatment.

Mechanical preparation was done using the ProTaper Next rotary system and a standardized irrigation protocol for the standardization of the procedure. The (NiTi) rotary instruments were chosen because they result in minimal debris extrusion compared to the stainless-steel hand K-files due to their design and motion [35, 36].

Vertical compaction techniques and ADSEAL were applied for the three groups since the obturation material mass becomes more homogeneous and adapts better to root canal irregularities compared to cold lateral compaction (CLC). Moreover, CLC is time-consuming, causes voids in the obturation mass, and increases the risk of vertical root fracture [37, 38].

In this study, 2780 nm Er,Cr:YSGG was used at a power of 1.25W. Er,Cr:YSGG has been used to clean the root canal system at output powers ranging from 1 to 3W [39, 40]. However, increasing the power above 1.5 W might potentially cause thermal damage. Moreover, high power Er,Cr:YSGG laser has limited usefulness in removing the smear layer because the structural damage that it causes in the dentinal structure in itself might become a source of smear layer formation [40, 41].

Diode Laser at a power of 1W in a continuous mode was used in this study. The continuous mode ensures equal distribution of light along the surface of the root canal and enhances the regeneration of periapical tissue. Continuous mode at a power higher than 1.5W is not advisable in the root canal because of the heat damage that it may cause [1, 9].

Following the methodology of Godbole et al.[42] in vitro study, the root canals were irradiated from the apical to the coronal portion, in a helicoidal movement touching the canal walls. This standardized protocol ensures uniform diffusion of laser light inside the root lumen and reduces heating of dentin, thereby avoiding damage to the surrounding periodontal tissues. This protocol improves the reduction of microbial load as shown also by Garcez et al.[43].

The total time of radiation was 40 s. It was divided into four times of 10 s irradiation and three times of 10 s pause between the lasing cycle. Each irradiation had a rest period of 10 s to avoid the temperature raise above the 7 °C threshold of periodontal tissues. Studies [15, 44] evaluating the rise in temperature on the external root surface during intracanal irradiation using Diode lasers have recorded temperature rise in the range of 1–7 °C. Since the threshold bone necrosis temperature for periapical structures is 47 °C for 1 min [45] this temperature rise is well below the danger threshold. Moreover, Al-Karadaghi et al. [23] evaluated in vitro the effects of dual wavelength (2780 nm Er,Cr:YSGG and 940 nm diode) laser on temperature changes during laser-assisted root canal treatment and concluded that within the parameters studied, the dual-wavelength laser did not lead to unfavorable thermal changes on the external root surface.

In this study, the bacterial count method was used to measure the viable cells in CFU/mL- × 10*(colony-forming units per milliliter). Some studies [46, 47] have shown that the culturing method demands a microbiological facility in close proximity to the dental office to ensure that the bacteria do not die in transit. To avoid this problem, root canal samples were cultured within four hours in the microbiology department after sample collection. Many advanced techniques have been proposed to evaluate the bacterial load of root canals, including flow cytometry, PCR, and others. Esterela et al. [48] compared the results obtained from PCR and culture techniques; the same results were obtained from both techniques despite the higher sensitivity of the PCR technique.

In the current study, we assessed aerobic and anaerobic bacteria. As the periapical infection is a polymicrobial disease, the aerobic culturing technique alone is not sufficient to reflect the microbiologic status of the root canal system [1]. Thus, the total aerobic and anaerobic count of bacteria were counted following the same methodology of Garcez et al. [47].

Several techniques have been described to obtain a representative sample of root canal bacteria. Orstavik et al.[49, 50] used reamers. However, in most of the vivo studies [1, 50, 51] root canal samples were acquired with paper points, as in this study.

In the conventional group, 5 ml of 2.5% sodium hypochlorite for 1 min together with 5 ml of 17% EDTA for 1 min was used in group 1 for disinfection and has resulted in 57.6% mean overall bacterial reduction. Similarly, Abbaszadegan et al. [52] Laukkanen et al. [53] and Rubio et al. [54] concluded that conventional endodontic treatments have proved to be insufficient to lower the detection limits of endodontic pathogenic microorganisms, with success percentage not exceeding 62% to 83%.

Superficial bacteria have shown a total bacterial reduction of 57.6% in the conventional group. However, an inferior rate of bacterial reduction is expected in the deeper layers of dentin because microorganisms can invade dentin up to a depth of 1100 µm [55]. While a 6% solution of NaOCl for 20 min at 45°C cannot penetrate the dentinal tubules more than 300 µm [56].

In the dual laser Er,CrYSGG/Diode group, a total bacterial reduction of 99.97% for the aerobic bacteria and 99.97% for the anaerobic bacteria was found in the S2 samples. Similarly, Aksoy et al. [57] evaluated the smear layer removal efficiency of Er,Cr;YSGG in vitro. They concluded that Er,Cr:YSGG laser application is more efficient than NaOCl irrigation in removing the smear layer. Montero et al. [58] also compared EDTA and Er,Cr:YSGG laser in vitro regarding debris and smear layer removal. They reported that the laser showed greater cleaning efficacy than EDTA and concluded that combining the laser with EDTA improved the cleanliness even better, as the effect was accumulative.

Moreover, Erben et al. [20] evaluated in vitro the bactericidal potential of 2780-nm Er,Cr:YSGG and 940-nm diode lasers. They concluded that the wavelength combination of Er,Cr:YSGG and 940-nm diode laser is safe and highly effective than either laser alone and is comparable to needle irrigation with sodium hypochlorite and EDTA.

Merigo et al. [59] evaluated the bactericidal effect of Er,Cr:YSGG laser irradiation alone and when associated with NaOCl irrigation on endodontic biofilm in freshly extracted single-rooted human teeth. They concluded that Er,Cr:YSGG laser has better decontamination capacity when used in conjunction with NaOCl irrigation. However, the present study demonstrates that optimum root canal disinfection could be achieved without NaOCl.

The combined (Diode/EDTA) group S2 sample resulted in a cumulative total bacterial reduction of 99.93% for aerobic bacteria and 99.94% for anaerobic bacteria when compared to the initial S1 sample. Similarly, Beer et al. [60] reported an average bacterial reduction of 98.66% in vitro using 940-nm diode laser and saline. Moreover, Morsy et al. [1] found that intracanal irradiation in vivo with Diode laser together with the application of NaOCl and EDTA resulted in a bacterial reduction of 99.73% for aerobic and 99.9% for anaerobic bacteria.

Masilionyte et al. [21] compared the outcomes of laser-assisted endodontic treatment/ retreatment using a 940-nm diode laser and EDTA versus the conventional protocol of NaOCl and EDTA in teeth with apical periodontitis. They concluded that the 940-nm diode laser-assisted endodontic protocol is an effective replacement to conventional treatment. The advantages of the Diode/EDTA protocol included decreased consumption of chemical irrigants and systemic antibiotics as well as promoting faster healing of periapical lesions.

In the present study, the conventional NaOCl/EDTA group resulted in a total bacterial reduction of 63.57% for aerobic bacteria and 58.79% for anaerobic bacteria, while the combined Diode/EDTA group S2 sample resulted in a total bacterial reduction of 99.93% for aerobic bacteria and 99.94% for anaerobic bacteria when compared to the initial S1 sample. Opposingly, Sohrabi et al. [17] found that the NaOCl eradicated 99.87% of the bacteria and showed a significantly enhanced antibacterial impact compared to the 980-nm diode laser, which resulted in 96.56% bacterial eradication. This difference from our results may be due to the fact that they tested the pure antibacterial effect of Diode laser not in conjunction with Er,Cr:YSGG laser or EDTA that aids in the removal of the smear layer. The promising results observed using laser-assisted disinfection warrant further investigation against ultrasonic activated irrigation [29] to establish their relative efficacy and identify potential niche applications for each [30]. Future studies are also warranted to evaluate the efficacy of Er,CrYSGG/Diode and EDTA/Diode in multirooted teeth with smaller clinical anatomical diameters. This will address potential limitations identified in the present investigation and broaden the generalizability of the findings.

## Conclusions

In this study we evaluated in vivo the bactericidal efficacy of three disinfection protocols for endodontic treatment of single-rooted teeth with apical periodontitis. The results indicated that both dual laser Er,CrYSGG/Diode and combined laser EDTA/Diode groups provide superior bactericidal effect compared to the conventional NaOCl/EDTA group.

## Data Availability

The datasets generated during and/or analyzed during the current study are available from the corresponding author upon reasonable request.
